# Ephaptic entrainment in hybrid neuronal model

**DOI:** 10.1038/s41598-022-05343-3

**Published:** 2022-01-31

**Authors:** Gabriel Moreno Cunha, Gilberto Corso, José Garcia Vivas Miranda, Gustavo Zampier Dos Santos Lima

**Affiliations:** 1 Departamento de Física Teórica e Experimental, Universidade Federal do Rio Grande do Norte, Natal, RN 59078-970 Brazil; 2 Escola de Ciências e Tecnologia, Universidade Federal do Rio Grande do Norte, Natal, RN 59078-970 Brazil; 3 Departamento de Biofísica e Farmacologia, Universidade Federal do Rio Grande do Norte, Natal, RN 59078-970 Brazil; 4 Instituto de Física, Universidade Federal da Bahia, Salvador, BA 40170-115 Brazil

**Keywords:** Computational biophysics, Mathematics and computing, Computational neuroscience

## Abstract

In recent decades, there has been a growing interest in the impact of electric fields generated in the brain. Transmembrane ionic currents originate electric fields in the extracellular space and are capable of affecting nearby neurons, a phenomenon called ephaptic neuronal communication. In the present work, the Quadratic Integrated-and-Fire model (QIF-E) underwent an adjustment/improvement to include the ephaptic entrainment behavior between neurons and electric fields. Indeed, the aim of our study is to validate the QIF-E model, which is a model to estimate the influence of electric fields on neurons. For this purpose, we evaluated whether the main properties observed in an experiment by Anastassiou et al. (Nat Neurosci 14:217–223, 2011), which analyzed the effect of an electric field on cortical pyramidal neurons, are reproduced with the QIF-E model. In this way, the analysis tools are employed according to the neuronal activity regime: (i) for the subthreshold regime, the circular statistic is used to describe the phase differences between the input stimulus signal (electrode) and the modeled membrane response; (ii) in the suprathreshold regime, the Population Vector and the Spike Field Coherence are used to estimate phase preferences and the entrainment intensity between the input stimulus and Action Potentials. The results observed are (i) in the subthreshold regime the values of the phase differences change with distinct frequencies of the input stimulus; (ii) in the supra-threshold regime the preferential phase of Action Potentials changes for different frequencies. In addition, we explore other parameters of the model, such as noise and membrane characteristic-time, in order to understand different types of neurons and extracellular environment related to ephaptic communication. Such results are consistent with results observed in empirical experiments based on ephaptic phenomenon. In addition, the QIF-E model allows further studies on the physiological importance of ephaptic communication in the brain, and its simplicity may open a door to simulate the ephaptic response in neuronal networks and assess the impact of ephaptic communication in such scenarios.

## Introduction

Understanding the interplay between mind and brain is one of the most challenging endeavors in science^1,2^. Discoveries in neurosciences have provided a unique insight through which we can observe the complex dynamics of the brain^3–6^. An important phenomenon in neuroscience studies involves neuronal communication^7–9^. Nerve cells communicate in various ways, via the exchange of small molecules and ions, as in the case of electrical and chemical synapses, or exclusively via electric fields. Communication made exclusively through electric fields is called ephaptic entrainment^10–16^.

The neuronal ephaptic entrainment is a communication known for several decades^12,14^. However, its physiological action and function are not very clear to this day^11,17^. Despite the lack of clarity regarding the physiological function of ephaptic entrainment, there are empirical studies that indicate a role for ephaptic entrainment in synaptic plasticity^18^, in the synchronization of neuronal activity by geometrical disposition^16,19–23^, in addition to the relation between ephaptic entrainment and neuronal dysfunctions, such as epilepsy and Parkinson disease^24–26^. In recent decades, advances in interdisciplinary science areas could only be achieved through appropriate mathematical approaches. In this sense, the physical sciences have contributed to the field of neuroscience in the study of biological phenomena and in the development of analytical tools to better understand experimental results^8,16^. However, analyzing physical phenomena requires sophisticated mathematical techniques, which include, among others, non-linear dynamics^27^, stochastic differential equations^28,29^, maximum entropy via microstates^30^, recurrence analysis^31^ or computational mathematics^32,33^. Thus, modeling is an essential component of scientific construction and support in understanding the behavior of nature in the most diverse areas of science. At this point, physical science can assist neuroscience in the elaboration and improvement of theories that involve, for example, extracellular electric field phenomena with the aid of mathematical models^7,17,34^.

Among several well-known neuronal simulation models^35^, the Integrate-and-Fire types are a wide family that originate from the model proposed by Lapicque in 1907^8^. This linear model uses a very simple circuit, but with great application in the neuronal area. Despite this, it is known that the relation between the total current of membrane ions and the voltage of a neuron membrane is not linear^35,36^. Thus, it is necessary to think of a generalization of the model proposed by Lapicque—which is not only done for the mathematical motivation of having a richer dynamic behavior—but also for the biological reasons mentioned above^36^. In this context, the Quadratic Integrate-and-Fire model (QIF)—a nonlinear model—was chosen because it comes from the analysis of the dynamic space of equations describing different types of excitable membranes^37^. Despite all that, the QIF was not designed—and until now not even used/applied—to simulate the case of ephaptic neuronal communication.

### Ephaptic quadratic integrate-and-fire model

The so-called integrate-and-fire models constitute a class of simple models of neurons that capture two basic elements of neuronal excitability: passive integration of subliminal inputs (below the threshold), and generation of identical pulses when the voltage reaches the trigger threshold. The QIF is a non-linear model because the relation between the total ionic current of the membrane and the voltage of the membrane of a neuron is non-linear (revealed by experimental studies). The quadratic integrate-and-fire model (QIF) is a model of neuronal dynamics, having two interesting characteristics, simplicity and low computational cost^38^. The QIF was proposed by Ermentrout^37^ and became very useful for the simulation of cortical neurons, as it shows the bifurcation of the saddle-node in the phase space^38^. The QIF model equation is given by:

1 $$\begin{aligned} C_{m}\frac{dV_m(t)}{dt} = \frac{(V_{m}(t)-V_{rest})(V_{m}(t)-V_{tresh})}{R_{m}(V_{tresh}-V_{rest})} + \left[ I(t) \right] \end{aligned}$$

and, if $V_{m} \ge V_{peak}, V_{m} = c$. In Eq. (1), $C_m$ is the membrane capacitance, $V_m$ the membrane potential, $V_{rest}$ the rest potential, $V_{tresh}$ the threshold of excitation value, $R_m$ the membrane resistance and *I*(*t*) the leak current across the membrane.

To adapt the QIF model to the ephaptic entrainment (QIF-E) we decompose the current [*I*(*t*)], as follows: $I(t) = I_{ephap}(t) + I_{0}$, where $I_{0}$ is a constant current (inside membrane observed in the empirical study^11^) to differentiate regimes: subthreshold ($I_{0} = 0$) and suprathreshold ($I_{0} \ne 0$). The electric potential is a physical magnitude that is useful when computed between two points. The law of Ohm, for instance, is always estimated with the potential difference between the terminals of a resistor. The estimated electric potential in our methodology is computed with help of the Ohm’s law across cell membrane. That means, the ephaptic current, $I_{ephap}(t)$, that crosses the membrane is estimated by the Ohm’s law $I_{ephap}(t) = \frac{V_{in}-V_{out}}{R_{m}}$. In the absence of the electrode, we have $V_{in} = - 65$ mV and $V_{out} = 0$ mV (grounded brain hypothesis^39^). When the electrode is turned on, the $V_{out}$ will be given by the Holt and Koch equation using the approximation of the electrical potential induced by a point spherical current source^7,15^. Indeed, our approach is in agreement with references^11,40^, given that the proposed model uses an extracellular potential derived from the solution of the Poisson equation for a current source inserted in the Ohmic conducting medium. Therefore, our approach implicitly considers the electrical potential gradient in the extracellular medium.

2 $$\begin{aligned} I_{ephap}(t) = \frac{-[I_{out}(t)+\epsilon (t)]}{4\pi \sigma _{out}rR_m}, \end{aligned}$$

where $\epsilon (t)$ is a noise added to the current, and *r* the distance between the neuron and the current source (input stimulus = $I_{out}$). The expression (2), plus the $I_{0}$ constant current, was replaced in the QIF expression (1), to provide the new the Quadratic Integrate-and-Fire Ephaptic model (QIF-E):

3 $$\begin{aligned} C_{m}\frac{dV_m}{dt} = \frac{(V_{m}-V_{rest})(V_{m}-V_{tresh})}{R_{m}(V_{tresh}-V_{rest})} + \left[ - \frac{I_{out}(t)+\epsilon (t)}{4\pi \sigma _{out}rR_m} + I_{0} \right] . \end{aligned}$$

Thus, we arrive at the adjusted equation to model the ephaptic entrainment in the QIF. The parameters used in this work are shown in the Table 1. The computational work was performed with the Euler method^36,41,42^ in MATLAB [see Supplementary Information for QIF-E step-by-step code].

**Table 1:** Cell membrane biophysical parameters employed in the simulation of the quadratic integrate-and-fire model.

Quantity	Value	Description	References
$V_{rest}$	− 65 mV	Rest potential	^16,43^
$V_{tresh}$	− 55 mV	Excitation thresholds	^16,43^
$C_{m}$	$1.10^{-2} \; {\text {F}}/{\text {m}}^{2}$	Membrane capacitance	^10^
$V_{peak}$	+ 55 mV	Peak value	^43^
c	− 70 mV	Hyperpolarization constant	^43^
$\sigma _{out}$	0.29 $\Omega ^{-1} {\text {m}}^{-1}$	Conductance of extracellular space	^11,44^
*r*	50 $\upmu$m	Distance between current source and the point of $V_{out}$	^11^
$R_m$	$2.10^{-1} \Omega {\text {m}}^{2}$	Resistance of the neuronal membrane	^10^

**Figure 1:**
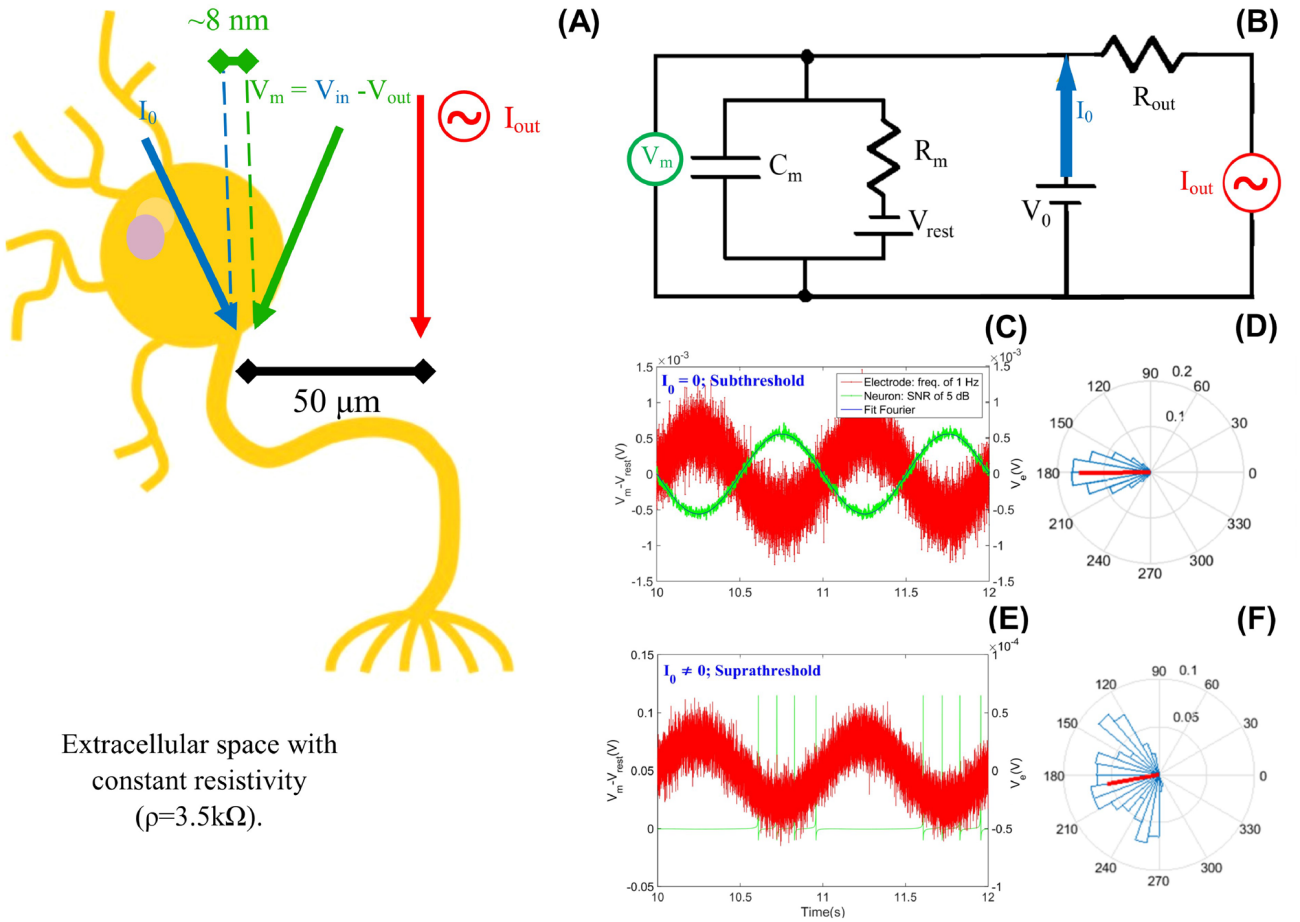
Schematic drawing of the cell membrane and its representative RC circuit for the Integrate-and-Fire quadratic model with ephaptic entrainment (QIF-E). Simulation of ephaptic neuronal entrainment via hybrid neuronal model. (**A**) Schematic drawing of the experience equivalent to the simulation. Two electrodes on the neuronal membrane provide the membrane potential (blue and green). The external electrode produce an oscillatory electric field via input stimulus ($I_{out}$ in red). The intracellular electrode (blue) can inject a constant current ($I_0$), differentiating the two simulation regimes: Subthreshold ($I_0 = 0$) and Suprathreshold ($I_0 \ne 0$). (**B**) RC circuit representing the QIF-E ephaptic model [see Eq. (3)]. (**C**) In subthreshold regime the input stimulus was represented in red and the frequency is 1 Hz and SNR of 5 dB, and the model response is in green. In blue, we see the signal filtered by the Fourier method, with the most intense frequency in the response signal. (**D**) Circular statistics of the phase differences between the input stimulus and the model response, calculated using the Hilbert transform method. The medium vector (red) and the classes of the circular histogram—dispersion—in blue. (**E**) In suprathreshold regime, the input stimulus is in red, the frequency used is 1 Hz and SNR of 5 dB. The model’s response indicates that spikes occur only at a certain stage of the stimulus signal. (**F**) Vector population data from (**E**).

In order to assess whether the proposed QIF-E model describes the characteristic phenomenology of the ephaptic entrainment—verified empirically^11^—we analyze the neuronal activity for subthreshold and suprathreshold regimes (see Fig. 1). So, we induce or not a constant current inside the membrane ($I_0$) producing or not spikes in the neurons [see Eq. (3)]. The subthreshold regime is adapted by the absence of the constant current, $I_0$ while the suprathreshold regime has the constant current $I_0$ different from zero.

Finally, for completeness, we present a control response model that corresponds to the absence of ephaptic signal. We developed a control response to compare with the QIF-E model. The control response is simulated using the standard QIF model, the results are show in Supplementary Informaton with the proper statistical analysis.

### Subthreshold statistical methods

In the subliminal regime ($I_0 = 0$), a noise intensity range is chosen (between 2.5 dB and 160 dB) and added to the input stimulus signal (the external electrode source = $I_ {out}$ ). The noise is added to simulate the electric fluctuation present in the extracellular space in between the electrode source (input stimulus) and the membrane (response stimulus); we use a signal-to-noise ratio (SNR, in decibel). The choice of the noise is made in order to approximate the data simulated to the QIF-E of the empirical data. We tested an entrainment of noise values to add to the external signal ($I_{out}$) (see Fig. 1).

To verify the effect of ephapticity in the model, we measure the phases of two signals ($V_m$ and $I_{out}$) and check whether the difference between the phases remains constant, via circular statistics. To fit the intensity of the ephaptic response, the simulated neuron signal ($V_m$) employs a Fourier filter. The filtering is considered only the first harmonic of the series (see Fig. 1C in blue, fitting the signal in green). To verify the phase difference between oscillatory signals, it is necessary to get the instant phases from those signals. To perform this task, we use the Hilbert transform by MATLAB. The CircStats^45^ properly represent the phase data. With the circular statistics, we estimated means and deviations of the phase difference between the input stimulus and output of the QIF-E model response.

### Suprathreshold statistical methods

In the suprathreshold regime ($I_0\ne 0$) (see Fig. 1E) the analyzes are focused on the relation between input stimulus signal (external electrode) and spikes (membrane response). To perform these analyses, the frequency, noise, and amplitude of the input stimulus signal are varied. Relation between spikes and input stimulus signals are analyzed using specific tools: the Population Vector (Fig. 1F), the Spike Triggered Average, and the Spike Field Coherence.

The Vector Population tool was developed to quantify spike’s angular preference using phase information^43,46,47^. In this way, the Hilbert transform supplies the instant phases of the input stimulus signal based on the spikes phase positions. The circular statistic provided the spike membrane preference related to input stimulus (see Fig. 1F).

The Spike Triggered Average (STA) is a specific tool to calculate the mean profile to occur a spike in a neuron related to the preference input stimulus phase^48^. In this estimation it is necessary two signals: an input stimulus signal and the membrane potential signal (model response). To obtain the STA one should take slices, $l_{i}$, of the input stimulus interval around the spikes instants in the neuron signal^49^. The slices, $l_{i}$ are chosen with a time window defined by simulation conditions. In the present work, the temporal window adopted to obtain the STA is $\frac{1}{f}$ for *f* the stimuli frequency.

Finally, the Spike Field Coherence (SFC) is a tool that measures how strong is the synchronization between a stimuli signal and a spike train, based in the STA analysis^11,50^. The SFC it is defined between 0 (without signal synchrony) and 1 (totally synchronous signal)^50^. The calculation to SFC is performed by the expression^51^ $SFC = \frac{\Psi (STA)}{\frac{1}{n}\sum ^{n}_{i=1}\Psi (l_{i})}$, where $\Psi ()$ is the power spectrum, STA is the Spike Triggered Average; the mean of the power spectrum estimated in the $l_{i}$ slices is the Spike Triggered Power (STP)^51^.

## Results

### Subthreshold regime

**Figure 2:**
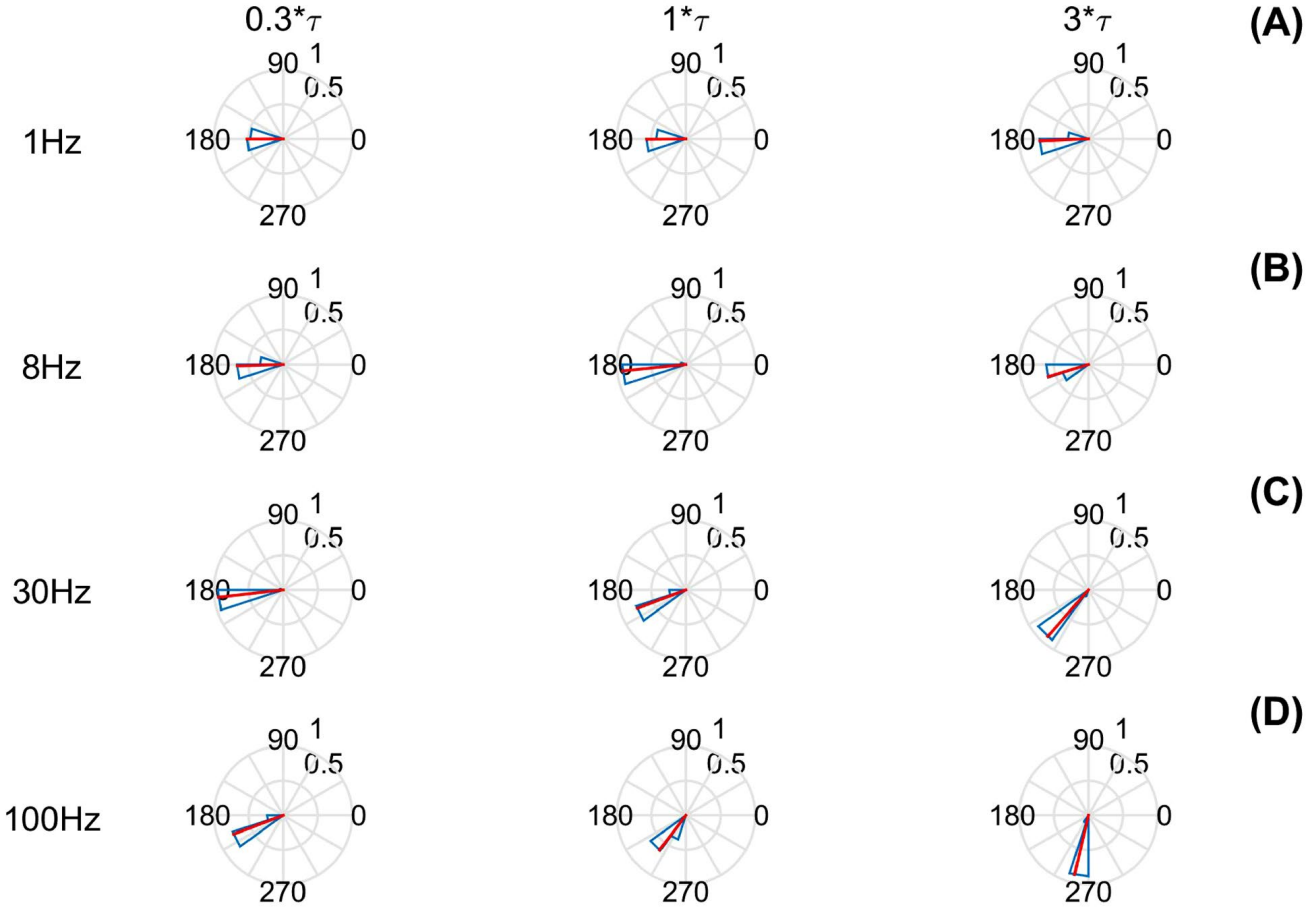
Subthreshold circular statistics for different parameters of characteristic times and frequencies. The characteristic time of the neuronal membrane has a defined value based on the experimental data present in the literature. Such values represent an increase (greater than 1) or a reduction (less than 1) of the LFP-type response speed model characteristic of ephaptic entrainment. In addition, we show how the various values of the frequency parameters of the input signal induce a phase difference. The columns show the circular statistics for a membrane: 0.3 times, 1 times, 3 time what is reported in the literature, with the associated frequencies (In the lines (**A**) 1 Hz, (**B**) 8 Hz, (**C**) 30 Hz, and (**D**) 100 Hz). In all results we chose the fixed amplitude (100 nA) and noise (20 dB) intensities that best suit the results of the experimental results. To guide the eyes and reference the intensity of the statistical value of the phase difference (red), all graphs show, on the right and above, two numbers (between 0 and 1) related to the radius size of the inner and outer circle, respectively.

In the subliminal regime, we explore several parameters within the proposed model following empirical studies, such as the frequency and the oscillation amplitude of the input stimulus signal, the noise, and the characteristic time. In Fig. 2 are shown the circular statistics of the phase difference for the subliminal regime, for a range of characteristic-times (0.3*$\tau$ to 3*$\tau$) and distinct frequencies (1 Hz, 8 Hz, 30 Hz and 100 Hz) of the input stimulus signal. Furthermore, we use noise and amplitude of the input stimulus signal fixed at 20 dB and 100 nA, (for a complete study of the parameters employed in the simulations of the ephaptic entrainment, see Supplementary Figs. 1 to 4).

Figure 2 display the phase difference (circular statistics) between input stimulus signal and membrane potential signal. To analyze the effect of the input stimulus signal oscillations in the neuron membrane response, four input signal frequencies were chosen—((A) 1 Hz, (B) 8 Hz, (C) 30 Hz, (D) 100 Hz)—the same frequencies as the analyzed empirical study^11^. Thus, we observe a dependence between the frequency of the input stimulus signal and the membrane potential response. In this way, the subthreshold regime outcomes of the QIF-E model follow the analyzed experimental data^11^, as shown in Table 2.

In addition, in Fig. 2 we also show how the choice of the characteristic membrane time ($\tau$) affects the phase difference between the input stimulus signal and the membrane potential response. The characteristic time of the neuronal membrane ($\tau$) is shown in each column, to compare with the experimental data presented in the literature^11^. To validate our model, we tested $\tau$ around the standard value reported in the literature ($\tau = 2$ ms)^10^ and multiply it by an arbitrary factor around the value ($0.3\tau$ to $3\tau$) of the model response to LFP-type stimuli characteristic of the ephaptic entrainment.

The values of characteristic time for a membrane are chosen as having: 0.3 times, 1 time, and 3 times the speed (for more analysis of time characteristic values and phase difference see Supplementary Figs. 3 and 4). Knowing that a fast membrane response is associated with a small characteristic time value ($\tau$) and that a slow membrane response is linked to a large characteristic time value ($\tau$), we observe that, for a fast membrane ($0.3\tau$ column), all frequencies produce an answer close to $180^\circ$ in phase difference. This characteristic is lost when we make the model slower ($3\tau$ column), mainly for higher frequencies (> 30 Hz), indicating that the model according to the desired electrophysiological characteristics for a given neuronal membrane.

To finish the study of the subthreshold regime, we analyzed two other parameters of our model: (i) the noise intensity and (ii) the signal amplitude ($I_{out}(t)$), both results are shown in the Supplementary Figs. 1 and 2. These results show that as noise increases (independent of the frequency), the circular statistics become more dispersed around the mean, but the phase difference does not change as observed in Supplementary Fig. 1 in Supplementary Information. Likewise, we observe that the model responds to different input stimulus signal amplitudes with different response amplitudes, but this change in its input stimulus intensity does not alter the phase differences either (see Supplementary Fig. 2 in Supplementary Information). These outcomes are expected because in the expression of, the difference in membrane potential ($V_m$) is directly proportional to the intensity of the input current ($I_{out}(t)$). Therefore, in this study, we take a current amplitude at 100nA and noise at 20bB to better fit the results of the subliminal experimental results and do not influence the phase difference between the electrode and the neuron signals^11^. Table 2 summarizes the data from the QIF-E model that best fits the empirical data^11^. This table shows the errors made by the QIF-E in estimating the subthreshold phase difference.

**Table 2:** Results of subthreshold empirical phase differences, and phase differences obtained by the QIF-E model. The last column contains the relative errors. Model data configuration of 100 nA, 20 dB and $\tau$.

Frequency (Hz)	Empirical^11^ (Grad)	QIF-E (Grad)	Error (%)
1	$190^\circ$	$180^\circ$	5.0
8	$188^\circ$	$187^\circ$	1.1
30	$210^\circ$	$201^\circ$	4.0
100	$231^\circ$	$233^\circ$	0.8

### Suprathreshold regime

For the supra-threshold regime, Eq. (3) is used, in which the constant current, different from zero $I_0$, plays the role of a non-ephaptic stimulus applied to the model. This extra stimulus inside the membrane is necessary since the ephaptic communication studied in this work does not have enough intensity to produce spikes in the QIF-E model, here we also compare our results with the empirical experiments in the current literature^11^. To treat the data obtained in this regime, three different analysis tools are used: the population vector tools; Spike Triggered Average (STA), and Spike Field Coherence (SFC). For a more extensive study of these parameters related to ephaptic entrainment, see Supplementary Figs. 5 to 8.

**Figure 3:**
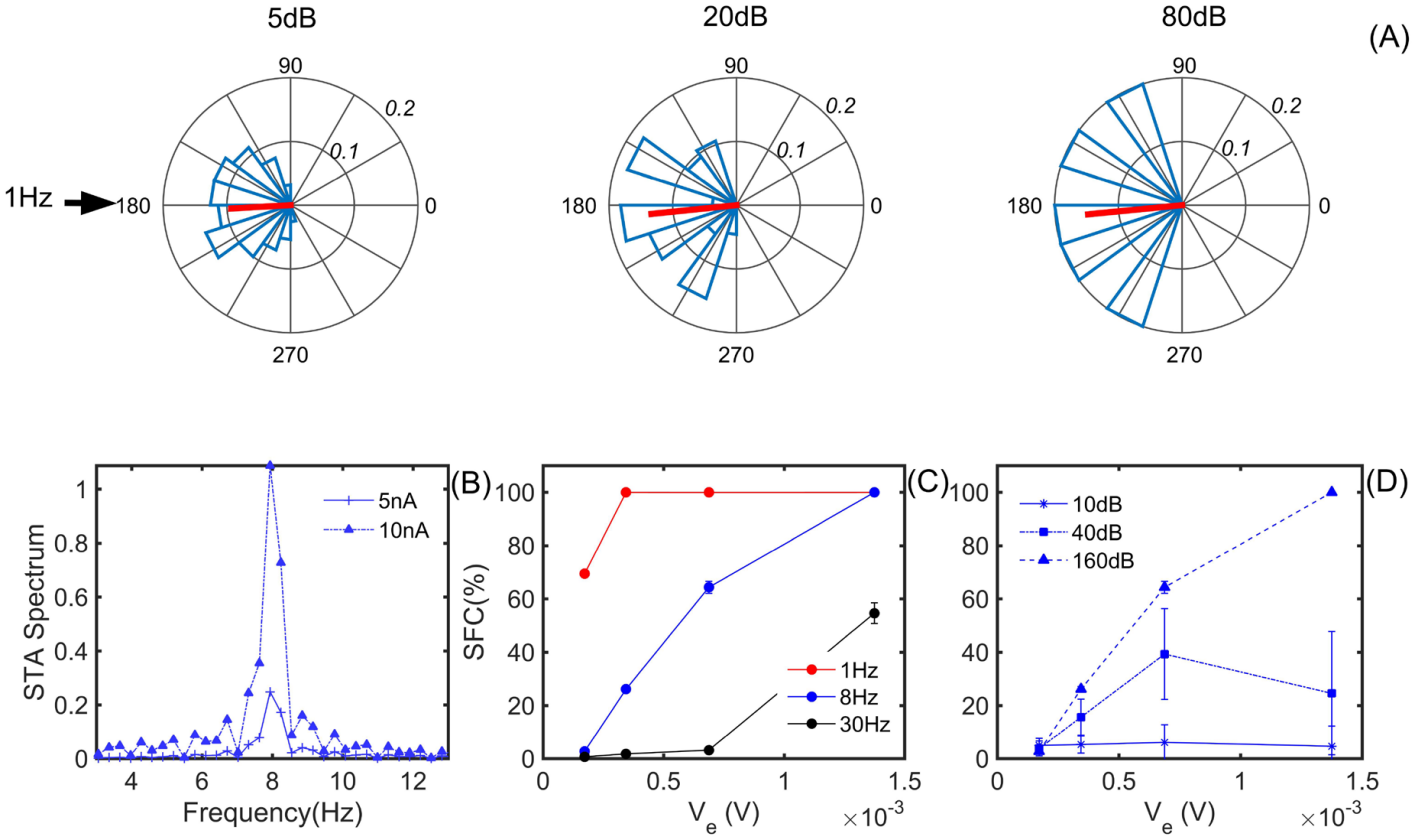
Results of the suprathreshold regimen. In panel (**A**) we show the population vector (spike phase preference) for a 1 Hz frequency and 10 nA amplitude of external stimulus. The noise does not change the direction of the population vector (for complete analysis with different frequencies, amplitudes and noises see Supplementary Figs. 5 and 6 in Supplementary Information). In (**B**) we observed that the intensity of the STA spectrum, given a frequency (8 Hz), is directly related to the signal intensity (5 nA and 10 nA). For this test the peaks occur with a frequency equal to that of the supplied stimulus, of 8 Hz. The outcomes for 1 Hz and 30 Hz, with 1.25, 2.5, 5 and 10 nA were observed in Supplementary Fig. 7 in Supplementary Information. (**C**) The SFC (entrainment intensity) results indicate that the higher the frequency of the stimulus signal (30Hz—in black), the less intense is the entrainment between the peaks and the external stimulus. Otherwise, for the frequency equal to 1 Hz, the SFC assumes a high value (in red). In panel (**D**) the SFC, for 8 Hz, were calculated for different noise intensities (10 dB, 40 dB and 160 dB) in the external signal. For the same analysis for 1 Hz and 30 Hz, see Supplementary Fig. 8 in Supplementary Information. Our results are similar to empirical outcomes.

Figure 3 shows the results of the supra-threshold regime. In panel (A) we observe the population vector for a 1 Hz frequency stimulus. Note that, like the subliminal regime, noise does not change the direction of the population vector, but it can change the distribution of the preferred phases for the model’s spike. Thus, we chose a fixed noise in the input stimulus signal of 20dB for the analysis of other parameters and tools. For a complete analysis of varying noise and intensities, see supplementary material. In (B) we observed that the intensity of the STA spectrum, given a frequency, is directly related to the signal intensity (1.25 nA, 2.5 nA, 5 nA, and 10 nA). For this test, it is noticeable that the STA peaks occur with a frequency equal to the frequency of the input stimulus signal, which in this case is 8 Hz.

In general, what is categorically observed in the STA and in its frequency spectrum, is that the input stimulus signal that produces the occurrence of spikes coincides with the provided input stimulus signal. This input stimulus profile is lost when increasing the noise intensity in the input stimulus, indicating again that the proposed QIF-E entrainment model is sensitive to noise and can reduce the ephaptic communication. Panel (C) shows that the SFC calculated for the QIF-E model indicates that the higher the frequency of the input stimulus signal (30 Hz—in black), the less intense is the entrainment between the peaks and the input stimulus. Otherwise, for the frequency below 1 Hz, the stimuli assume a high value. In panel (D) the SFC calculated for different noise intensities in the input stimulus signal, the previous observations regarding the intensity of the entrainment between the input stimulus signal and the model’s response.

From Fig. 2 we see that the intensity of the input stimulus signal ($I_{out}$), as well as its frequency and noise, are factors that influence ephaptic entrainment in the suprathreshold regime. Based on the SFC analysis, we noticed that the modeled entrainment is sensitive to the intensity of the input stimulus, the frequencies of the input stimulus, and also to the intensities of noise. This low entrainment intensity between the input stimulus signal and the model’s suprathreshold response is most likely responsible for the characteristics observed in the population vector. All of these observations agree with the suprathreshold behaviors from empirical results.

Table 3 shows the relative errors between the population vector phase values of the QIF-E model and the population vector phase values given by the empirical study^11^. It is important to note that the angular deviation measured in population vectors via QIF-E are large enough to reach the empirical values and reduce errors.

**Table 3:** Results of suprathreshold empirical population vector phases, and population vector phases obtained by the QIF-E model. The last column display the relative errors. Model data correspond to the configuration of 1 Hz, 10 dB.

Stimulus amplitude (nA)	Empirical^11^ (Grad)	QIF-E (Grad)	Error (%)
2.5	$250^\circ$	$200^\circ$	20
5	$242^\circ$	$194^\circ$	20
10	$241^\circ$	$183^\circ$	24

According to Anastassiou et al.^11^, there is an average behavior involving ephaptic communication relating to the two studied regimes. Thus, under the same initial conditions (parameters and variables) in the suprathreshold regime as the input stimulus current of the external electrode increases, the membrane potential phase preference tends to the same value observed in the subthreshold regime of the phase difference between the input stimulus and the membrane response. Thus, there is a neurological response—relating the two regimes—in the empirical study that is similar in the QIF-E model. This result is possibly related to the membrane’s time to recharge and fire again as it receives an external field stimulus intensity. Thus, Table 4 shows this difference—phase difference (Table 2) and phase preference (Table 3)- –between the results of the two regimes: sub- and supra-thresholds, respectively, for both analysis: QIF-E model and empirical outcomes. Thus, increasing the current amplitude shows a decrease in the difference between these two results, decreasing the error between them. The QIF-E model obtained a result of this behavior (increase in amplitude decrease in relative error) similar to that found in the empirical study.

**Table 4:** Results of suprathreshold empirical population vector phases, and population vector phases obtained by the QIF-E model. The last column contains the relative errors. Model data corresponds to the configuration: frequency of 1 Hz and noise of 10 dB.

Stimulus amplitude (nA)	Empirical^11^ (Grad)	Error (%)	QIF-E (Grad)	Error (%)
	Supra − sub = $\Delta$	$\Delta$/sub	Supra − sub = $\Delta$	$\Delta$/sub
2.5	$250^\circ - 190^\circ = 60^\circ$	32	$200^\circ - 180^\circ = 20^\circ$	11
5	$242^\circ - 190^\circ = 54^\circ$	28	$194^\circ - 180^\circ = 14^\circ$	8
10	$241^\circ - 190^\circ = 51^\circ$	27	$183^\circ - 180^\circ = 3^\circ$	2

## Discussion and conclusion

To validate our QIF-E model, we test the characteristics of ephaptic communication in individual neurons, corroborating the empirical data from Anastassiou et al.^11^. In the current panorama of the study on ephaptic entrainment, some models consider the dynamics of communication activity in neurons. In fact, most models simulate the ephaptic effects caused in the propagation of the nerve impulse along nerve fibers and axons^16,17,52^, using a cable theory approach^34^. However, such models are not the best options since they often use continuous neural models, which are, in general, more costly than integrate-and-fire models^53^. Besides that, the use of continuous models can be an arduous and thankless task in the case of simulations with numerous neurons. To overcome this obstacle, the development and use of an integrate-and-fire (hybrid) model with the ephaptic entrainment concept may be computationally more suitable than other neuronal models.

The main characteristics of dynamic behavior from the neuronal ephaptic entrainment of the QIF-E model are presented and compared with the empirical study^11^; such as the existence of an anti-phase, total or partial, between the input stimulus signal and the membrane response. The noise is implemented in our model so that the result becomes closer to the empirical experiment, once it better simulates the extracellular environment between the external electrode (input stimulus) and the neuronal membrane. We notice that the phase difference is not altered by noise (see Supplementary Information). The analysis of different intensities of input stimulus signal and the respective phase difference responses are shown in the supplementary material as well. The variation in the intensity of the input stimulus signal shows that the phase difference present between signals in the QIF-E model does not depend on the intensity of the input stimulus signal. This is due to the fact that the phase shown in Eq. (1) depends neither on the intensity nor on the current nor on the potential difference applied to the circuit. These results reinforce the adequacy of this model to simulate the characteristics of ephaptic communication already observed in the literature^11,17^.

In addition to the aforementioned characteristics, it is possible to generalize the model so that not only the phenomenology of the sub-threshold regime is compatible with the experimental results. The generalization of the model can be done with variations in the value of the model’s response time parameter, $\tau$, and should be done based on the physiological aspects of the neuronal membranes since the $\tau$ is related to the capacitive and resistive properties of the neuronal membrane. Thus, Fig. 2 shows the phase difference, via circular statistics, when we vary the characteristic time of the model associated with the neuronal membrane. The characteristic time of the membrane causes a phase difference between this time and the electrode signal. Therefore, the phase difference is associated with the response time of the model to the input signal and the response of the neuron.

In Fig. 3 we highlight the evidence of the preferential phase for the population vector for the generated spike, regardless of the noise inserted in the signal. The results of the SFC show the dependence with the increase of the input stimulus frequency and are consistent with empirical measures^11^. Further, the faster the oscillation (100 Hz) of the stimulus signal, the less intensely the spike is related to a preferential phase. So, we conclude that the QIF-E model adapted for an ephaptic current adequately simulates the neuronal ephaptic entrainment, since the subliminal and supra-threshold properties of the model are consistent with the results observed in the studies.

The monopolar approximation of the electric dipole field employed in the QIF-E model is valid for scales smaller than $150\;\upmu \hbox {m}$, as discussed in Ref.^39^. This typical size delimits the region where the monopolar approximation of the dipole field presents an error lesser than 1$\%$. A more accurate dipole treatment is employed when the distances are above $150\;\upmu \hbox {m}$. In this situation the monopolar approximation becomes inadequate, which is the case, for instance, of the experiment presented in Rebollo et al.^54^. This experiment showed evidence of an endogenous field effect and also designed a model that includes ephaptic interactions using dipole–dipole like interactions. On the other hand, it is noteworthy that the experimental evidence presented by Anastassiou et al.^11^, shows a monopolar decay of the electric potential (Fig. 1C of^11^). Thus, the approximation to the monopolar electric potential, for dimensions smaller than $150\;\upmu \hbox {m}$, is adequate for the case of a cell and a close electrode in the extracellular space. The QIF-E model suggested here follows the parameters (physical properties) used in the empirical experiment by Anastassiou, thus, the monopolar interaction was considered for the simulations. In opposition, the Rebollo’s experiment, which considers a tissue with dimensions greater than 150 $\upmu$m (around 0.5 mm), the dipole model is necessary.

For the understanding of ephaptic communication, it is essential to study the impacts of this communication on the neuronal cell and its implications on the central nervous system. For this reason, a range of ways to study this communication at various levels is essential, from simulations of the propagation dynamics of spikes and how this dynamic is affected by the ephaptic entrainment^7,15,16,20,52^, even the simulation of coupling in sets of neurons (network)^17^, as our model makes possible. So, the formulation of the QIF-E model made in the present work makes it possible to raise new hypotheses about the function of coupling in healthy nervous tissues, as well as the impact of coupling in dysfunctions already related or not to ephaptic communication. A question raised by the model is that modifying the electrophysiological properties of membranes can affect the quality of ephaptic entrainment ( the control response model, that means, the case ephaptic off, does not show spike drag and spike preferential phase typical of the entrainment phenomenon - see the Supplementary material). This modification was shown in the variation of the characteristic time ($\tau$) could be—for example—related to demyelination of the neuronal membrane^24^, which causes a change in the capacitance of the membrane, or to a change in the resistance of the neuronal membrane, caused by biological variations between individuals, or even by genetic dysfunctions.

It is noteworthy that modeling a scenario close to reality requires simulations with numerous neurons involved. In this case, the use of continuous models becomes unfeasible, leaving room for hybrid models, such as the models of the integrate-and-fire^36^ family. Therefore, the study of the impact generated by ephaptic entrainment in simulations with several cells is favorable to the QIF-E model with ephaptic entrainment, proposed in the present study, which is the main motivation of the model. As already mentioned, the QIF-E model proved to be able to simulate the ephaptic characteristics observed experimentally in cortical pyramidal neurons^11^. Since the QIF model is widely used to simulate pyramidal cortical neurons^38^, the QIF-E model becomes a natural candidate for studies on the ephaptic effects in this class of neurons. Finally, whether due to the success of the QIF-E in simulating ephaptic entrainment or the possibilities provided by the model, it is evident that the QIF-E with ephaptic entrainment is promising for the study of ephaptic communication and its repercussions on nervous tissue.

## Supplementary Information

Supplementary Information.
