# Supplementary material for: Ephaptic entrainment in hybrid neuronal model
Source: Sci Rep. 2022 Jan 31;12:1629. doi: 10.1038/s41598-022-05343-3 (PMC8803837; doi:10.1038/s41598-022-05343-3)
Supplement: Supplementary file 1 — Supplementary Information. [file 41598_2022_5343_MOESM1_ESM.pdf]

Supplementary Information

Title: Ephaptic Entrainment in Hybrid Neuronal Model.

Authors:
Gabriel Moreno Cunha,
Gilberto Corso,
José Garcia Vivas Miranda,
Gustavo Zampier Dos Santos Lima.

1 Ephaptic-off *versus* Ephaptic-on models

We developed a control response model to compare with the QIF-E model. The control response is simulated with the standard QIF model, as show below. This point becomes evident when we look at the QIF-E model equation (1):

$$C_m \frac{dV_m}{dt} = \frac{(V_m - V_{rest})(V_m - V_{tresh})}{R_m(V_{tresh} - V_{rest})} + \left[-\frac{I_{out}(t) + \epsilon(t)}{4\pi\sigma_{out} r R_m} + I_0 \right] \tag{1}$$

For the control group we have $I_{out} = 0$ and the analogous equation for the non-ephaptic case becomes:

$$C_m \frac{dV_m}{dt} = \frac{(V_m - V_{rest})(V_m - V_{tresh})}{R_m(V_{tresh} - V_{rest})} + \left[-\frac{\epsilon(t)}{4\pi\sigma_{out} r R_m} + I_0 \right] \tag{2}$$

The equation (2) basically corresponds to the standard QIF model. To highlight the effect described by our proposed model, some extra simulations comparing the control group (Ephaptic Off) and the QIF-E (Ephaptic On) are shown in figure 1.

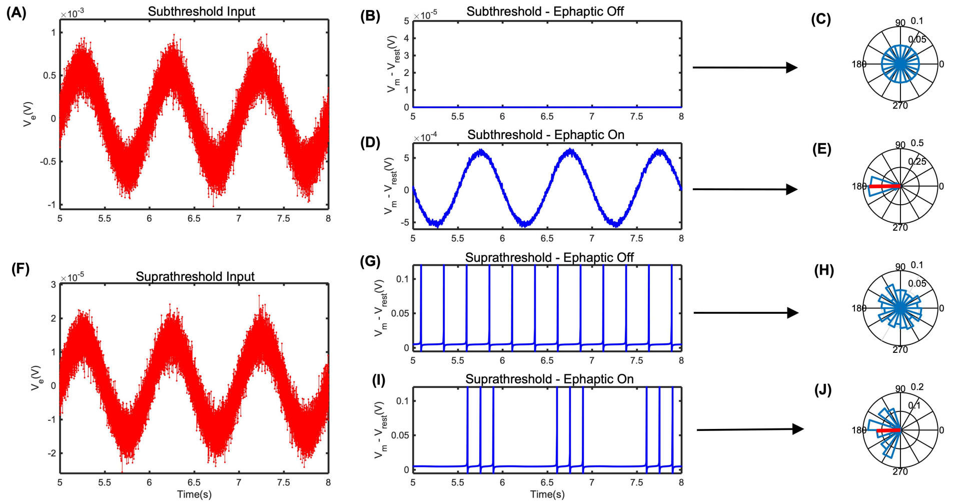

Figure 1: Control and Ephaptic groups. (A) Signal of the electric potential for the sub-threshold regime: 1 Hz, 100 nA, 10 dB. (B) Control group (ephaptic off) and (C) its circular statistics. (D) QIF-E (ephaptic on). (E) Its circular statistics. (F) Signal of the electric potential for Suprathreshold regime for 1 Hz, 5 nA, 10 dB. (G) The control group (ephaptic off) and (H) its circular statistics. (I) The QIF-E (ephaptic on) and its (J) circular statistics.

Indeed, in figure 1 we compare the control group (QIF) and the ephaptic group (QIF-E), for the sub- and supra-threshold regimes. For both regimes, the circular statistics show the emergence of phase preference when the ephaptic entrainment is on. This effect was also experimentally observed in Anastassiou et al.

Subthreshold Results

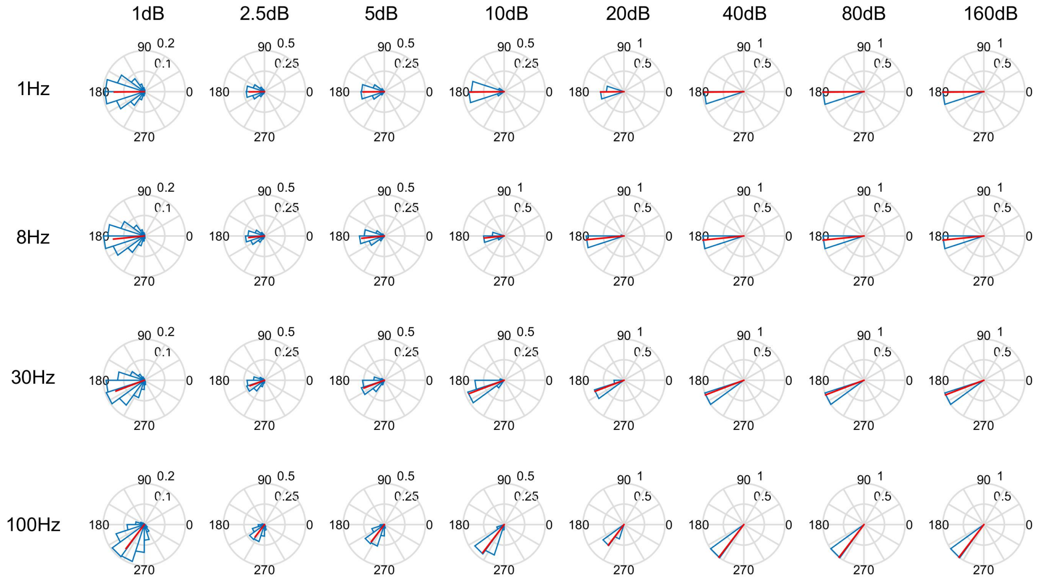

Figure 2: **The impact of noise on ephaptic coupling in the subliminal regime.** Here, we show how the injection of noise into the external electrode signal does not change the phase difference between the electrode signal and the membrane potential at different external signal frequencies. We use an input signal amplitude equal to 100nA fixed. Each column refers to a specific noise value 1dB, 2.5dB, 5dB, 10dB, 20dB, 40dB, 80dB, 160dB (SNR indicated at the top) against a frequency (1Hz, 8Hz, 30Hz, 100Hz) of the input signal (line). We observe that the ephaptic coupling does not have a correlation with the noise change, that is, the increase of the SNR does not alter the phase difference given a specific frequency. Equivalent results are observed in an empirical study.

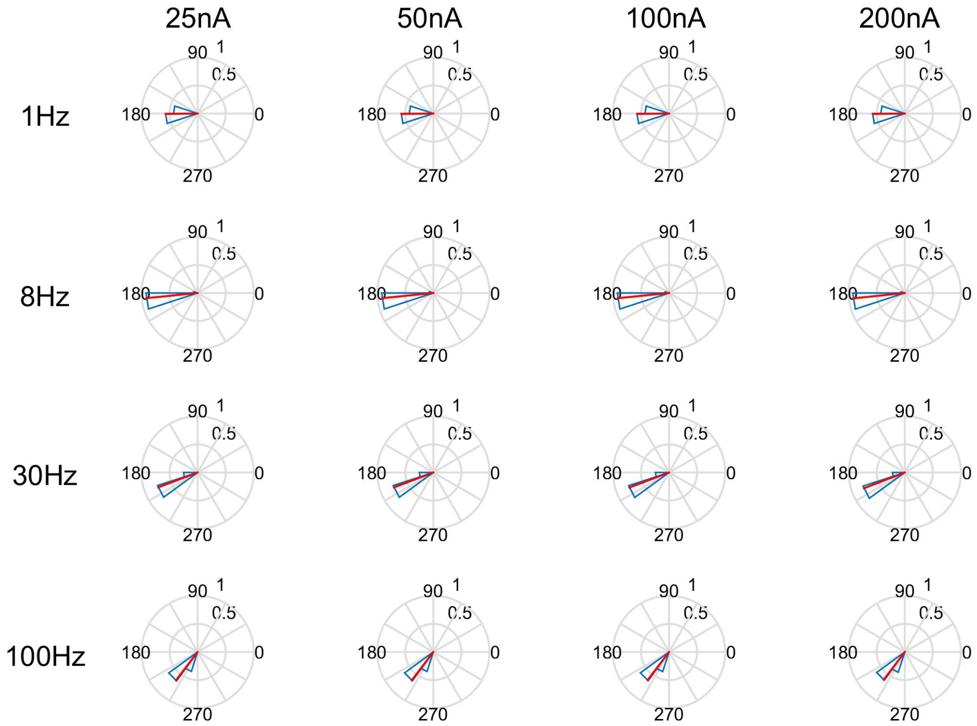

Figure 3: **The impact of external signal amplitude on ephaptic coupling in the subliminal regime.** Phase differences in the subthreshold regime are analyzed for different amplitudes of the external signal (column) against the different oscillation frequencies of the external signal (row). Here, we choose a noise equal to 20dB as a fixed input parameter. Each column refers to an external current amplitude value, I_{out}, 25nA, 50nA, 100nA, 200nA (indicated at the top) and each row refers to a specific frequency of the input signal (indicated at the side). The amplitude of the input signal does not change the phase difference between the input signal and the membrane potential for a specific frequency. Similar results are seen in an empirical study.

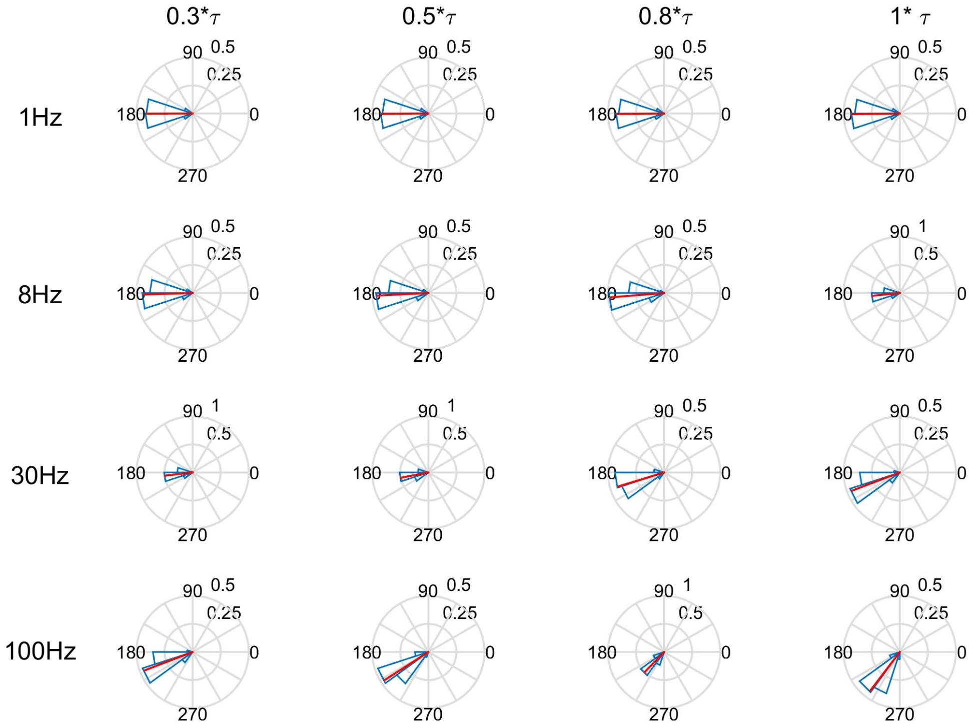

Figure 4: **Characteristic time impact on ephaptic coupling in the subliminal regime.** We use the fixed 20dB and 100 nA inputs. Each column refers to a membrane response value, τ, multiplied by a factor less than one (indicated in the top) and each row refers to a specific frequency of the input signal (indicated at the side).

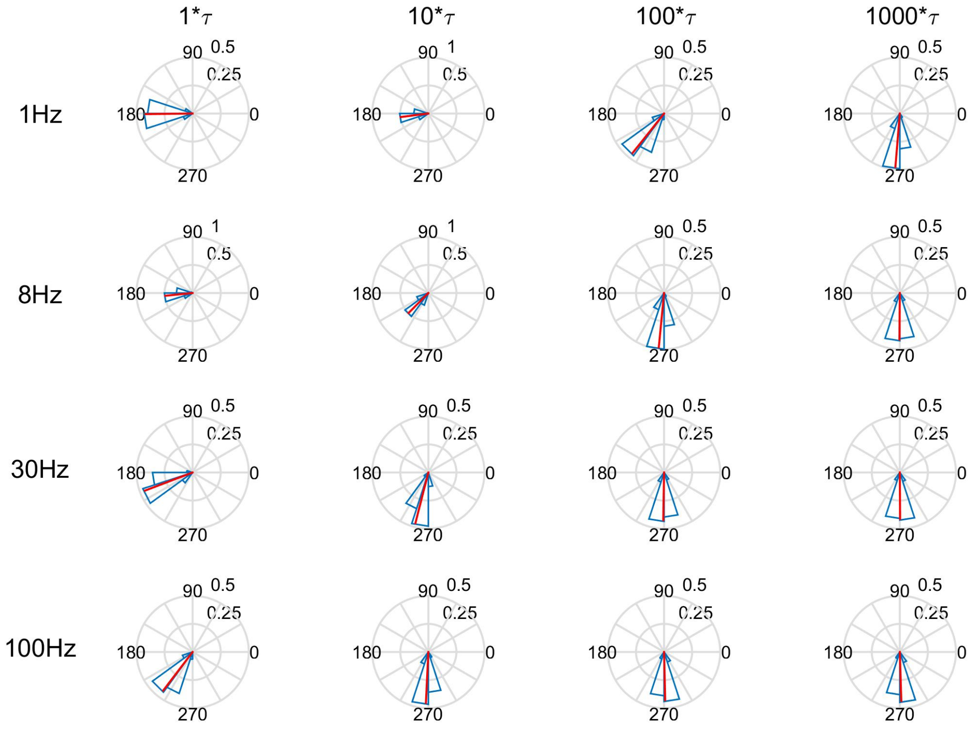

Figure 5: **Characteristic time impact on ephaptic coupling in the subliminal regime.** We use the fixed 20dB and 100nA inputs. Each column refers to a membrane response value, τ, multiplied by a factor greater than one (indicated at the top) and each row refers to a specific frequency of the input signal (indicated at the side). Values much larger than one order of magnitude may not represent physiological characteristics. However, these values help in the process of understanding how the model works. Since the QIF-E is valid, supra physiological values of membrane time can help to understand, in an approximate way, anomalous physiological processes and their necessary conditions.

Suprathreshold Results

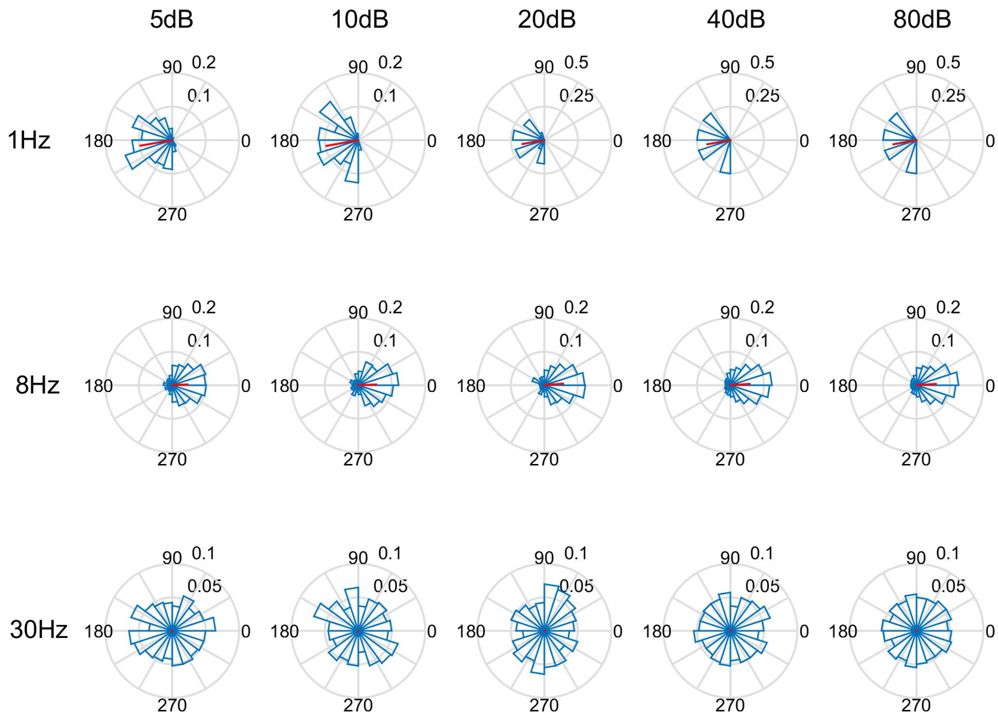

Figure 6: **Preferential phases of the spikes indicated by the population vector.** In this figure the 5nA signal was chosen. Here it is possible to observe that the noise (5, 10, 20, 40 and 80 dB, SNR indicated at the top) applied to the external signal is not able to affect the phase preference of the spikes of the QIF-E model. As well as in the case of subthreshold phase differences, here the existence of a frequency dependent phase (1, 8 and 30 Hz, indicated on the left side) of the external signal is observed here. This result is in line with empirical observations. In our study, for the supra-threshold regimen, four external signal amplitudes, 1.25, 2.5, 5 and 10 nA were used

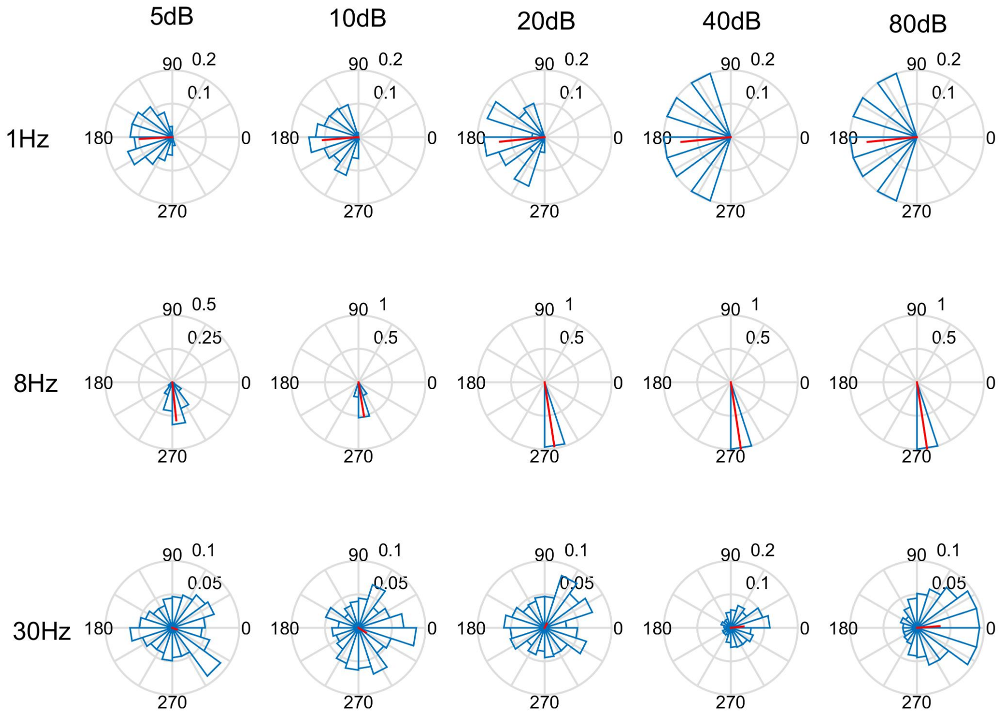

Figure 7: **Preferential phases of the spikes indicated by the population vector.** In this figure the external signal amplitude of 10nA was chosen. When comparing this figure with the Figure 6, it is possible to note that the preferential phase of the spikes changes in relation to the previous result, in the 8Hz external frequency. This change is due to the increase suffered by the coupling, shown by the results of SFC as a function of external signal strength. The preferential phase shift was similarly observed empirically.

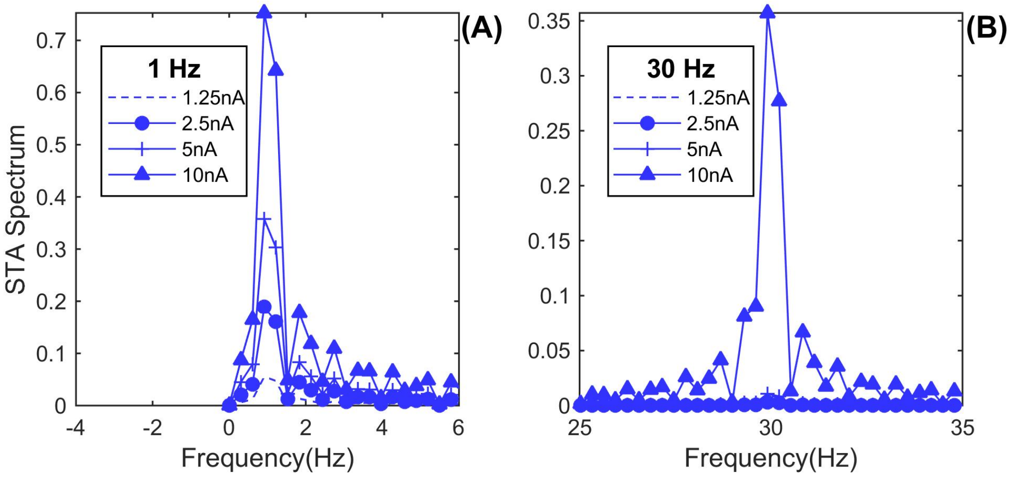

Figure 8: **Emerging STA frequency via STA Spectrum.** STA as an average stimulus linked to a higher occurrence of spikes can provide valuable information. When obtaining the STA power spectrum, it is possible to observe if there is a privileged frequency in the spike generation process. Our results indicate that the privileged frequencies coincide with the frequencies of the external signal, as we observed here in the case (A) 1 Hz, and (B) 30 Hz (For all external current intensities $[I_{out}]$ worked, 1.25, 2.5, 5 and 10 nA). Also note that the intensity of the peak of the spectrum is pronounced dependent on the strength of the external signal. Equivalent results were seen in the experiment. Furthermore, noise is not able to modify the behavior of the STA Spectrum. Here, an SNR of 5 dB was used.

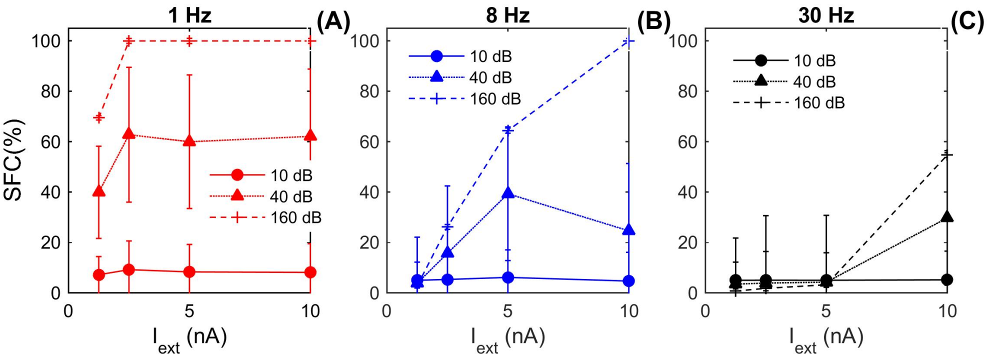

Figure 9: **Mean intensity spike-phase coupling.** The strength of the coupling between the spike and its preferred phase in the external signal is measured by the SFC. The graphs from (A) to (C) are for the external signal frequencies of 1, 8 and 30 Hz, respectively, worked in the supra-threshold regime. With these figures, it is possible to observe that the noise, despite not being able to modify the preferred phase of the spikes of the QIF-E model, alters the intensity of the phase-spike coupling. This result has two important impacts. The first one is associated with model validation, since we can change the noise to adjust the coupling intensity values according to the physiological values seen and already documented. The second impact of this result is linked to possible changes in the extracellular environment caused by some neurodegenerative diseases.
